# A qualitative exploration of the prospective acceptability of the MiDerm app; a complex digital intervention for adults living with skin conditions

**DOI:** 10.1111/bjhp.12778

**Published:** 2025-01-05

**Authors:** Rachael M. Hewitt, Carys Dale, Catherine Purcell, Rachael Pattinson, Chris Bundy

**Affiliations:** ^1^ School of Healthcare Sciences Cardiff University Wales UK; ^2^ School of Dentistry Cardiff University Wales UK

**Keywords:** dermatology, digital intervention, qualitative research

## Abstract

**Objectives:**

Skin conditions carry a substantial psychological burden but support for patients is limited. Digital technology could support patient self‐management; we found preliminary evidence for the effectiveness and acceptability of digital psychological interventions for adults living with skin conditions. We have, therefore, developed a complex digital intervention called MiDerm with patients. This qualitative study explored the prospective acceptability of the complex intervention delivered via a smartphone application (app), and possible barriers and facilitators to use.

**Design:**

Qualitative research involving a hybrid inductive‐deductive approach. Data collection and analysis were theoretically informed by The Common‐Sense Model of Self‐Regulation, Theoretical Framework of Acceptability and the Capability, Opportunity, Motivation ‐ Behaviour Model.

**Methods:**

Eight synchronous online group interviews with 43 English‐speaking adults (≥ 18 years) with skin conditions. Data were analysed using Reflexive Thematic Analysis.

**Results:**

Three superordinate themes were generated: (1) *Patients' attitudes and concerns about the MiDerm app;* (2) *Need for personal competence, autonomy and relatedness for effective self‐management;* and (3) *Physical, psychological and social barriers to app use*.

**Conclusion:**

Adults with skin conditions, mainly those with vitiligo and psoriasis living in the UK, expressed the need for support to self‐manage the psychological aspects of their condition(s). The idea of a new intervention comprised of informational, emotional, behavioural and peer support, delivered via a smartphone app was welcomed and may be especially beneficial for specific patients. Identified barriers must be addressed to maximize engagement and giving users choice, flexibility and control is imperative to this. We have since developed the MiDerm app using these findings.

**Statement of contribution**

**What Is Already Known about this Subject?**

Skin conditions carry a significant psychological and social burden, but dedicated psychological support for this population is limited. In addition to this, health‐threatening behaviours, such as smoking cigarettes, are common in those with skin conditions. Our research indicates that digitally delivered interventions in dermatology may be useful for improving psychological outcomes and treatment‐related behaviours.

**What Does this Study Add?**

- The idea of a smartphone app providing psychological and behaviour change support was acceptable to adults with skin conditions.
- MiDerm was considered an appropriate adjunct to standard medical care that could facilitate self‐management and continuity of care.
- Barriers exist but respecting user autonomy and flexible use could facilitate engagement with MiDerm.

## INTRODUCTION

Skin conditions carry a substantial physical, psychological and social burden for patients (Cartron & Driscoll, 2019; Gisondi et al., 2023; Lee et al., 2023; Oliveira et al., 2015; Patel & Jafferany, 2020; Toussi et al., 2021). Our research shows that physical, psychological, social, financial and daily impacts are common across skin conditions, but the psychological and social impacts are most profound (Pattinson et al., 2022). Despite this, integrated, specialist psychological support and psychodermatology services are limited, which is a global problem in dermatology (All‐Party Parliamentary Group on Skin, 2020; Misery et al., 2023). Additional forms of support are, therefore, required to facilitate patient self‐management (NHS England, 2022).

Health‐threatening behaviours (e.g. smoking, drinking alcohol, low physical activity and poor sleep and diet) are common in people with skin conditions (Al‐Jefri et al., 2017; Massoud et al., 2021; Yeroushalmi et al., 2022; Zanesco et al., 2022). They can trigger and worsen skin conditions (Sawada et al., 2021) and increase associated cardiovascular disease (CVD) risk (Public Health England, 2017). Behaviour change plays an important role in skin condition management but patients generally receive generic lifestyle advice (Trettin et al., 2021).

Digital technologies offer a useful platform for supporting the self‐management of long‐term health conditions (van Gemert‐Pijnen et al., 2018). Our recent systematic review of 23 papers found that digitally delivered interventions in dermatology improved some psychological (i.e. knowledge of dermatological conditions and their management, mood, quality of life) and clinical (i.e. disease severity short‐term, the therapeutic relationship) outcomes (Hewitt, Ploszajski, et al., 2022), but most were web‐based, educational, condition‐specific and targeted treatment‐related and skin protection behaviours (Hewitt, Ploszajski, et al., 2022). Other studies have since shown promising results for digital intervention effectiveness, but are also limited to treatment adherence in people with eczema (Gudmundsdóttir et al., 2022; Santer et al., 2022) and skin protection behaviours in psoriasis (Cline et al., 2022) and xeroderma pigmentosum (XP); (Walburn et al., 2023), respectively. In addition to this, very few interventions exist for those living with rare skin conditions (Hewitt, Ploszajski, et al., 2022). Since our systematic review was conducted, other studies have been published on digital interventions in dermatology. However, little has changed since as the recent studies are all condition‐specific, with the majority targeting people (some including young people) usually with eczema (Andrade et al., 2023; Greenwell, Daniela, et al., 2022; Greenwell, Ghio, et al., 2022; Gudmundsdóttir et al., 2023; Kern et al., 2023; Kishimoto et al., 2023; Weigandt et al., 2023; Yasuda et al., 2023) followed by psoriasis (Brandl et al., 2022; Fortune et al., 2022; Garzorz‐Stark et al., 2021) and other conditions including XP (Walburn et al., 2023), acne (Ip, Muller, Geraghty, Rumsby, et al., 2021) and visible differences inclusive of some skin conditions (Norman et al., 2022; Zelihić et al., 2022).

Arguably, new digital interventions for common and rare skin conditions are needed, as are those which address changing modifiable health risk behaviours. MiDerm is intended for use across both common and rare skin conditions, differentiating it from existing condition specific interventions.

We also found preliminary evidence for the acceptability of digitally delivered interventions, although qualitative research was poorly reported and lacking, as was patient input to intervention development (Hewitt, Ploszajski, et al., 2022). Understanding the user perspective is key to developing complex interventions (Skivington et al., 2021) and qualitative research, which is generally limited in dermatology, should be prioritized (Foster et al., 2022) to ensure digital interventions are relevant for patients and work as intended (Hewitt & Bundy, 2021).

Incorporating the perspective of prospective users is the cornerstone of the Person‐Based Approach (PBA) to systematically planning, evaluating and implementing behavioural interventions supporting the self‐management of health and illness (Yardley et al., 2015). The PBA champions using qualitative research to better understand people's views and experiences to develop interventions that are appropriate, practical, meaningful and effective for them (Morrison et al., 2018).

We followed the PBA to co‐develop a complex digital intervention called MiDerm for adults living with skin condition(s). Following our systematic literature review (Hewitt, Ploszajski, et al., 2022), we conducted a qualitative semi‐structured group interview study aiming to determine the prospective acceptability of MiDerm and gather insights from the target group to inform its development, as well as exploring potential barriers and facilitators to use of the app.

## METHODS

### Design

Qualitative research involving synchronous, online, semi‐structured group interviews. Ethical approval was obtained from the School of Healthcare Sciences Research Ethics Committee (SREC: REC807).

### Participants

Adults (≥18 years) living with a skin condition(s) who could access the internet and spoke English sufficiently to take part in a group interview. There were no exclusion criteria.

#### Recruitment

The study was advertised on social media (Twitter, Facebook, LinkedIn and Instagram) by the research team and five patient organizations, including:

- The International Alliance of Dermatology Patient Organizations (aka GlobalSkin).
- The British Skin Foundation.
- Skin Care Cymru.
- The Psoriasis Association UK.
- Eczema Outreach Support.

These organizations also shared the study details via email, on their websites and in member magazines or newsletters.

Initially, participants were recruited on a voluntary basis and were enrolled to the study providing they had completed the online registration form and deemed eligible. As the study progressed, we priortised diversifying the sample and used demographic information to sample purposively thereafter. Purposive sampling was employed by sharing specific advertisements for demographics which were underrepresented in the current sample (e.g. male participants, people of colour). Recruitment ceased when no new information was emerging from the data.

### Materials

#### Online registration form

An online registration form, including participant information, an e‐consent form and a short demographic questionnaire, was created using the Jisc Online Survey platform.

#### Topic guide

A semi‐structured topic guide was developed, exploring the (1) impact and (2) self‐management of skin conditions, (3) existing types of support, and (4) the prospective acceptability of a digital app. Items were based on the study aims, the clinical and academic expertise of the research team, and relevant concepts from the Capability Opportunity Motivation‐Behaviour (COM‐B) model (Michie et al., 2011); and the Theoretical Framework of Acceptability (TFA); (Sekhon et al., 2017). Six members of our Patient and Public Involvement (PPI) group with eczema, rosacea, psoriasis and X‐linked ichthyosis reviewed the topic guide. Participants were told that the findings of the study would inform the development of a new smartphone application to support people with skin conditions by helping them to better understand their condition and live well in their own skin.

The current paper focuses on results pertaining to the prospective acceptability of MiDerm; results related to condition impact and management are reported elsewhere (Hewitt et al., 2024).

### Procedure

Recruitment spanned February 2022 to June 2022.

Eligible participants were invited to a scheduled group interview and asked to indicate their availability in a private Doodle poll sent via email. Participants were subsequently offered specific dates and times to select, and therefore group assignment was based on participant preference. Each participant received an email confirming the details of their group interview plus joining instructions. Group sizes were based on recommended participant numbers for focus groups in similar studies; up to 10 participants per group (Coulson, 2015).

The lead author (RH) conducted and audio recorded all interviews via Zoom. Use of the ‘raise hand’ function and chat box was encouraged. Audio recordings were transcribed verbatim by an external transcription service.

Reflections on the reflexivity of the researcher can be found in Material S1.

### Analysis

Data collection and analysis were concurrent; any topics that were discussed, which were not covered in the original topic guide, were incorporated and probed in later interviews.

Interview transcripts were anonymised and imported to NVivo 12 Pro. Qualitative data were analysed using Reflexive Thematic Analysis (Braun & Clarke, 2022). Chat box data were analysed along with interview transcripts. Data were analysed at the latent level from an essentialist/realist perspective to capture, contextualize and critically interpret the participants' personal views, experiences and ideas.

Two analytical frameworks were applied to the data. The TFA (Sekhon et al., 2017) was used to explore the prospective acceptability of MiDerm. The COM‐B Model (Michie et al., 2011) was employed to map perceived barriers and enablers to using the app against established drivers of behaviour. Further descriptions of the theoretical frameworks used in this study can be found in Material S 2.

Two authors (RH and RP) piloted the analytical frameworks on one transcript initially. Any discrepancies in the coding were resolved through discussion and revisions were made where necessary. In addition, as the TFA is traditionally used to investigate the acceptability of established healthcare interventions, we adapted the definitions of the seven concepts of the TFA to reflect the hypothetical nature of the proposed digital intervention (see Material S3). This process was repeated on other transcripts until the authors agreed the frameworks were comprehensive and applied consistently.

One author (RH) completed data familiarization and coding. Data were coded deductively to identify data supporting existing concepts within the analytical frameworks, and inductively to capture information that was separate from the frameworks but relevant to the study aims. Themes were derived inductively from the codes identified and agreed by the research team. Quantitative demographic data were imported to IBM SPSS Statistics 27 and descriptive statistics were calculated to describe the sample characteristics.

## RESULTS

Forty‐three people participated across eight group interviews. Group sizes varied, with the smallest group comprising of two participants and the largest involving seven participants. Most participants identified as female (69.8%), white ethnicity (83.7%), and lived in England, UK (67.4%). Whilst the location of participants was acknowledged, the variation was minimal and therefore there was little scope to derive conclusions based upon this. The average age was 44.63 (*SD* = 3.02). Most had vitiligo (27.9%) followed by psoriasis (25.6%) and were not affiliated with a patient organization (67.4%). The average number of years lived with a skin condition was 28.02 (*SD* = 15.92). Sample characteristics are presented in Material S4.

Six themes were derived from the data. This paper reports on three themes specific to app development. The titles of themes and sub‐themes related to this paper are as follows:

1. Patients' attitudes and concerns about the MiDerm app.
  1.1 App meets need and desire for support.
  1.2 Appropriateness.
  1.3 Concerns reflecting poor understanding of condition impact and the app's purpose
2. Need for personal competence, autonomy and relatedness for effective self‐management
  2.1 Understanding me.
  2.2 Understanding my condition.
  2.3 Monitoring physical and psychological factors.
  2.4 Understanding others
3. Physical, psychological and social barriers to app use.
  3.1 Personal choice and autonomy.
  3.2 Look and feel of the app.

The remaining three themes are reported elsewhere (Hewitt et al., 2024).

### Theme 1: Patients' attitudes and concerns about the MiDerm app

This theme supports the prospective acceptability of MiDerm, highlighting specific sub‐groups of patients who the app may benefit. It indicates that altering the beliefs adults hold about skin conditions could alleviate concerns about the appropriateness of the app.

#### Sub‐theme 1.1: App meets need and desire for support

Participants reported that the psychological burden of skin conditions was overlooked and believed MiDerm could be a convenient and reliable source of support for self‐management.

“That it's all in one place, would be useful, because you just don't have that at the moment. […] when you're given a new cream, like you search back twenty years […] You know, to have it all in an app and all the information up to date, current, easily accessible, would be ideal I think.” (Group 2, Psoriasis, England)

#### Sub‐theme 1.2: Appropriateness

Participants commented on the potential of MiDerm to provide continued care from childhood through to adulthood, but perceived the app to be more appropriate for adults with late‐onset skin conditions due to lesser time adapting to their condition, and children and young people (C&YP) who face added pressures during these early transitional life stages. Some believed the app could support younger people to form health protective self‐management habits including appropriate treatment adherence:

“… I think the more you get into something, the more it becomes a behaviour and you get more used to it. Especially with children with skin conditions, they don't like creaming, they don't like doing self‐care as such, but if you do something in the app… I don't know, they track it and it gets better, that might really help.” (Group 7, Lamellar Ichthyosis, Wales)

#### Sub‐theme 1.3: Concerns reflecting poor understanding of condition impact and the app's purpose

Whilst attitudes towards MiDerm were generally positive, some concerns were expressed, such as how the app would differ from existing health apps.

Most participants had no personal experience of psychological support and struggled to envisage how MiDerm would provide this. Some questioned how the app would meet varying levels of need. Signposting users to existing forms of psychological support was proposed as a useful addition.

“Great in principle, it depends on the content and how tailored it is to different skin conditions. Also wonder how suitable it would be for people with more substantial mental health problems e.g. major depression linked to their skin condition vs those with milder symptoms.” (Group 8, Multiple skin conditions, England)

Participants were unsure if, or how, the app could cater for different skin conditions; only two recognized that there are common types of impact across skin conditions.

“…although we all have different conditions, there was a lot of commonality and there's a lot of common ground…” (Group 1, Hidradenitis Suppurativa, Ireland)

These findings show that some people lacked a full understanding of skin conditions and their impact, and the intended purpose of the MiDerm app.

### Theme 2: Need for personal competence, autonomy and relatedness for effective self‐management

Self‐determination Theory (SDT); (Deci & Ryan, 1985) argues that behaviour is driven by the psychological need to feel personal competence, autonomy and relatedness. Despite low expectations of support, participants' ideas for the MiDerm app were aligned to SDT; they wanted to better understand their skin condition and its impact (competence) and how others self‐manage (relatedness) to support them to become a specialist in managing their skin condition (autonomy).

“It's just the fact that the App is dedicated, for people like us […] a massive part of that will be mental health, but also like, there is also the physical things that you go through, which is like, making sure that you're physically comfortable, and being put in touch with people, helps. Your three pillars, you've got the physical aspect, the social aspect and the mental health aspect.” (Group 4, Vitiligo, Scotland)

#### Sub‐theme 2.1: Understanding me

Participants were willing to learn about links between their skin condition, behaviour and self‐management strategies and MiDerm was seen as an appropriate platform to address these links.

“… often people in this, who are in this situation where exercise can help, they, they don't realise it, they don't see it. […] a lot of people here have said that exercise has helped them and a lot of people who have skin conditions probably wouldn't think of it, so just suggesting it, erm, just suggesting it to people that would be a positive thing…” (Group 8, Multiple skin conditions, Denmark)

Acceptance was deemed important for adapting to skin conditions. Incorporating compassion and strengths‐based approaches (e.g. body functionality or neutrality) was promoted. Whilst some had come to terms with their condition, others struggled with negative self‐perception but wanted support to overcome this.

“… and body functionality which is basically focusing on what your body can do, rather than just focusing on how it looks. There's lots of evidence that it can help people feel better about themselves and kind of increase positive body image, so shifting people's thinking away from, for example, what they don't like about their body, to thinking about what it can do…” (Group 8, Eczema, England)

Alexithymia, the inability to recognize and label emotional responses, is common in people with psoriasis (Panasiti et al., 2020). Participants felt MiDerm could offer a safe space for people to process, label and understand their emotions related to their condition, and deliver practical tips for managing difficult emotions. Opinions on evidence‐based techniques, such as mindfulness, were mixed. Negative views about the use of instructional language were expressed.

“I'm just also quite against the term ‘mindfulness’ […] I'm just quite wary of apps that are like: ‘Oh, you're head's really itching. So, why don't you go for a walk and it'll take your mind off it, or why you don't you do some meditation for 5 min, and then you'll feel great?’ I suppose it's just balancing that like non‐patronising aspect, but I do think it's a rally fantastic idea.” (Group 2, Seborrheic dermatitis & Psoriasis, England)

#### Sub‐theme 2.2: Understanding my skin condition

Participants wanted to understand the interplay between psychological processes and skin physiology. They reported frustration when health professionals imparted general lifestyle advice without explanation of skin‐related benefits. Participants wanted practical tips for coping and management and believed this information could increase awareness and knowledge, provide a useful steer for self‐management, and improve their personal sense of control.

“Control is a very important thing, it's one of the aspects I struggled with a lot […] educating myself about my condition has been empowering, and it's given me back some sense of control.” (Group 1, Hidradenitis Suppurativa, Ireland)

The provision of evidenced‐based information dispelling common myths about skin conditions was also a priority and was perceived to be an effective way to alleviate common concerns.

The Common‐Sense Model of Self‐Regulation (Leventhal et al., 1984) emphasizes the importance of illness beliefs in shaping emotional and behavioural responses. Although some participants were aware of causes of skin conditions, there was uncertainty around genetics and whether skin conditions were inherited. This uncertainty underpinned participants' concerns about starting a family, and the fear about their children inheriting their skin condition.

“I'm terrified of my daughter getting it, like how, how she would react. […] I would like to know if it could affect my daughter. So, it's that unknown.” (Group 4, Vitiligo, Scotland)

Female participants discussed the unpredictable nature of their skin conditions during key reproductive stages, including pregnancy, the menopause, and when using hormonal contraceptives. Knowledge about the link between the endocrine system and skin was lacking among participants and reportedly healthcare professionals also. One participant described being a resource for other women seeking advice, highlighting a need for evidence‐based guidance on female reproductive health and skin conditions.

“I've answered a lot of questions about pregnancy and ichthyosis […] I was just learning as I went […]. So, I can, you know, point them in the right direction, where to go for that. Um, and then I've had a few women that have ichthyosis, that have reached out, wanting to know like if their skin changes at all while they're pregnant.” (Group 3, Lamellar Ichthyosis, USA)

#### Sub‐theme 2.3: Monitoring physical and psychological factors

Participants felt a self‐monitoring function would allow identification of personal triggers and improved understanding of, and motivation for, behaviour change. They reported this could reduce the time and cognitive load of recalling their medical and treatment history in consultations, which could be months apart. They wanted to be able to share their data from the app with health professionals to facilitate meaningful discussions about their condition and its impact, and shared decision‐making.

“It can be really hard to remember [treatments and outcomes], and even when you're seeing a dermatologist and they've got all the information in front of them, they may not necessarily have comments about what has or hasn't worked, and it may not be your own view of what has and hasn't worked. […] If you can't get an appointment for ages, and then you're there, you've got 10 min. You can't remember your questions; you can't remember your history. Um, yeah, that could be helpful.” (Group 2, Psoriasis, England)

However, several participants relayed disadvantages that they had encountered using other health‐tracking apps, including health anxiety and increased pressure:

“I know there's quite a lot of, erm, research and things around like fitness apps and sometimes they're actually kind of encouraging like unhealthy behaviours or people becoming quite obsessed or, erm, if people susceptible to eating exercise disorders that can be problematic […] I think trying to make it useful but not trying to make it into one of those things where people feel like they have to be leading a certain lifestyle or doing a certain thing.” (Group 8, Eczema, England)

#### Sub‐theme 2.4: Understanding others

Social and peer support involves the exchange of knowledge and experiences among people with a specific long‐term health condition to aid adjustment, self‐management, and coping (Dennis, 2003). Participants saw peer support as a useful form of psychological support; they valued the idea of an app offering peer support to reduce isolation, facilitate meaningful discussions with similar others, and learn new self‐management and coping strategies.

“it's about knowing people that have, are living with the same condition […] It really touches home, because you think ‘Oh my God, yeah, that, that lady or that guy has just written about something that, that's exactly how I felt, and that's exactly how, and it just makes you feel like you're not the only one with this, you know, and there are millions of people out there, across the world, that are in exactly the same boat as you.” (G6, Vitiligo, England)

But potential disadvantages of online peer support were recognized, including poor regulation and the availability of inaccurate and potentially harmful advice, which could increase psychological burden.

“When I went onto some Support Groups on like Facebook and that. And they tell you like different things and they don't tell you that, or if you take like 20% like, Tretinoin for example, you're going to, you're going to be all scaly. So, yeah, just need to be aware, it just needs to be monitored, the people only share factual information and information that can't, wouldn't make somebody do it to burn their face and things like that, or skin, or anything.” (Group 6, Hyperpigmentation, Wales)

The provision of patient stories on coping with and self‐managing skin conditions was suggested as an appropriate alternative:

“Hear, hear to hearing inspirational stories from others. Patients listen to other patients more than anyone else.” (Group 1, Hidradenitis Suppurativa, Ireland)

### Theme 3: Physical, psychological and social barriers to app use

*Physical Capability*: The presence of physical symptoms (e.g. pain, impaired vision, sensitive skin or limited dexterity) were a perceived barrier to manually entering data into apps. Ease of use was deemed essential and prompting with basic questions and response options was recommended to minimize the burden. A few participants said the app should account for people living with multiple skin conditions.

“For me, I wouldn't want the app to be too much effort. I don't want to document things, just maybe click when I feel the need.” (Group 2, Vitiligo, England)

*Psychological capability*. Lack of emotional expression and use of avoidant coping strategies learned during childhood were discussed as barriers.

“… I'm a bit of a … kind of an ostrich. […] Er, I come from a family where we don't talk about things. Er, so it's never been a question, to talk about how I feel, or if I have a problem, or … So, I haven't really talked about it that hard, frankly.” (Group 3, Netherton Syndrome, Sweden)

Participants were sceptical towards corporate and pharmaceutical involvement in app development, and proposed endorsement from reputable health and charitable organizations as a solution.

“… [Moderator]: [participant] said ‘no links to big pharma’, [participant] do you want to say a bit more?” “[Participant]: … I think being suspicious of anyone who you think is trying to sell you medications [laughs]. Erm, you know, I think it's important the developers have integrity, erm, and you feel like they genuinely have like patients best interests at heart and, so I think it would be good if the skin charities were kind of able to endorse the content and say, you know, this is a trustworthy source […] I think would give confidence.” (Group 8, Multiple skin conditions, England)

There was a common belief that some people, particularly older adults, may lack the skills and confidence to use the app. Participants wanted a user‐friendly app with a simple interface that is easy to navigate.

“… I think of myself as being quite computer literate, but then I look at my grandchildren and I don't … I'm nowhere in their league. […] you mustn't always make the assumption that everybody that has a skin condition is young and has grown up using their thumbs for technology.” (Group 2, Vitiligo, England)

Participants recognized poor health literacy could hinder engagement and suggested using plain language, avoiding jargon, and incorporating visual content to improve understanding of the content. Translating the app into other common languages was also recommended to increase engagement globally.

*Social opportunity*: Participants alluded to the persisting public and self‐stigma surrounding skin conditions and seeking mental health support as a potential barrier.

“do people think that they go to mental support because they're going mad or something like that? And there's still a bit of a stigma, a stigma in my mind, certainly. But there may be a stigma in people's mind. It's a skin condition at the end of the day. Yes, it does affect you mentally.” (Group 6, Lamellar Ichthyosis, England)

The provision of psychological support via the app might provide an opportunity for adults to learn about skin conditions without having to ask for help.

Promoting the app through trusted sources (e.g. health providers and patient organizations) and app stores was seen as a way to increase awareness among prospective users and legitimize the app.

“I think going direct to the Organisations/Societies and ask them to put the App on their site(s) and recommending members to download it. Also, Doctor surgeries advertising it and all Groups associated with skin conditions.” (Group 6, Vitiligo, England)

*Physical opportunity*. Cost was identified as a potential barrier, although a few participants believed a small subscription fee might deter people with ulterior motives from accessing the app and increase user safety. Most believed MiDerm should be free to access given the extra expenses associated with managing skin conditions. Making MiDerm available on prescription was proposed to improve access among people on lower incomes.

“… even if it was quite cheap to buy, if people have got limited income or are spending money on quite a lot of other helps, then I think it [no cost] would open it up to more people.” (Group 1, Psoriasis, Wales)

*Reflective motivation*. Participants explained that their engagement with apps varied depending on day‐to‐day feelings, indicating low motivation could be a barrier to consistent use.

“I always start off really well with these tracking Apps and I am like the perfect student for the first three days and then I just am really bad at them…” (Group 5, Psoriasis, England)

Incorporating user feedback to ensure the content remains relevant and up‐to‐date was recommended to maintain motivation. Noticing improvements in symptoms was also a motivator for continued use.

### Sub‐theme 3.1: Personal choice and autonomy

Long‐term use seemed to depend on respecting the autonomy and preferences of users, and allowing flexible use depending on disease activity and severity.

“… if you think, yeah, I've kind of conquered it for a few months and then you kind of have to go back with your tail behind your legs and be like, oh it's come back again […] you may not be getting people using it, day in, day out, constantly, but it would be nice, if there's, if it's easy for people to take a break from it and then step back in.” (Group 5, Psoriasis, England)

Push notifications were discussed with mixed opinions. Frequent and instructional messages were described as frustrating. Participants emphasized the importance of push notifications serving to remind people about the app, instead of instructing them to use it. Generally, the app should not be prescriptive and should be flexible to suit the user. Allowing choice over the frequency of push notifications, and including motivational messages, was recommended to reduce the psychological burden and maintain engagement.

“I personally think that too many prompts and too many reminders could, could perhaps have negative connotations, because if you're already beating yourself up because, you know, you perhaps think you've done something that aggravated your skin condition […] it could have a negative downside, make you feel worse.” (Group 2, Vitiligo, England)

The consensus were comfortable inputting their personal data into the app, providing that data protection protocols (i.e. GDPR) are respected and data handlers are trustworthy. Offering the option to register anonymously under a username was suggested to alleviate potential concerns about user privacy and confidentiality.

“Yeah, if I thought that whoever was holding the information were reputable […] if it was a university, health organisation, someone like that, I'd have no problem whatsoever.” (Group 1, Hidradenitis Suppurativa, Ireland)

“I think some people wouldn't want to share information unless they were anonymous.” (Group 8, Multiple skin conditions, Denmark)

### Sub‐theme 3.2: Look and feel of the app

App design was important both for inclusivity and creating a positive user experience. Participants suggested including on‐screen captions and audio descriptions of content to improve accessibility for adults with impaired hearing or vision, sometimes resulting from skin conditions, such as Netherton Syndrome. Considering the colour scheme was advised to ensure appropriateness for people who are colour‐blind.

“I know within the Netherton syndrome community, most people have eye problems so bigger fonts is a great idea.” (Group 8, Netherton Syndrome, England)

Some existing apps reportedly resembled a ‘clinical’ environment and were not engaging. Participants wanted the colour scheme of the app to feel friendly, welcoming and calming. Participants requested light and pastel colours, mainly shades of blue or green, which were perceived to match those of existing apps in the health, well‐being and fitness space.

*“… but I do think that the idea of sort of looking at what some of the gyms do may not be a bad idea. […] It's all about, you know, let's, let's be positive and let's talk about food […] and you know, like pictures of people living their full life, which is what I suppose we're wanting to, to do here.”* (Group 6, Eczema, England)

## DISCUSSION

The idea of a complex behaviour change intervention delivered via a smartphone app was considered acceptable to participants with skin conditions. Participants desired an app that was evidence‐based to help them manage the psychological aspects of skin conditions. Desirable content included: high quality information on the links between skin conditions, mood and health behaviours, tips for trigger spotting, and established strategies for coping and self‐management. Participants recognized the potential of MiDerm to facilitate self‐acceptance, a sense of personal control, and enabling health behaviour change. MiDerm was considered a useful adjunct to existing care that could serve as a clinical tool and improve shared decision‐making during consultations and a sense of continued care in‐between appointments. Participants emphasized several barriers to access and engagement and suggested how the app's content, functionality and design could overcome these.

### What this study adds

There is a lack of rigorous qualitative research in intervention development and evaluation (Hewitt, Ploszajski, et al., 2022), and dermatology more generally (Nelson, 2015; Pascual et al., 2023). To our knowledge, this is the only qualitative study to explore and evidence the *prospective* acceptability of a complex digital behaviour change intervention in this area, setting an example for other dermatology researchers.

Participants in this study felt that MiDerm could help facilitate continuity of care from childhood to adulthood. We recognize the role of peer support in adjusting to having a skin conditions but online support requires better regulation to ensure the accuracy of information and safety and well‐being of users, as reported previously (Petukhova et al., 2020). Our participants suggested patient stories may be an acceptable alternative that could offer some of the same benefits as online support groups (e.g. learning ways of coping and managing), whilst offsetting the issues of safety, credibility and trustworthiness and the psychological burden these carry for some users.

In their study of photoprotection behaviours, Walburn and colleagues (Walburn et al., 2023) found that people with XP attributed behaviour change to greater awareness, increased motivation, goal setting, and habit formation following use of a complex, personalized and multi‐modal intervention. Similarly, we found the provision of evidence‐based material and approaches might increase knowledge, behavioural intentions, habit formation and promote health behaviour change, in a larger sample representing a range of skin conditions.

Whilst health tracking devices can increase motivation for change in people with long‐term health conditions (Birkhoff & Smeltzer, 2017), they can also increase health anxiety in some (Rosman et al., 2020). Our sample suggested the importance of self‐monitoring physical and psychological symptoms and triggers but advised caution with a tracking feature to avoid increasing psychological burden.

The COM‐B model (Michie et al., 2011) was used to help to focus discussion on acceptability, and participants suggested how MiDerm's content, functionality and design could facilitate engagement, increase capability, provide opportunities and motivate people for change. Ensuring content is relevant to the target audience (Walburn et al., 2023; Zucchelli et al., 2021) and up‐to‐date were deemed important (Teasdale et al., 2018), as was ease of use for people lacking digital skills and those with limited dexterity (Sangers et al., 2021; Zucchelli et al., 2021). Flexible use was a key facilitator (Zucchelli et al., 2021).

Text messages can act as useful reminders to reinforce new health behaviours (Walburn et al., 2023). Our participants expressed mixed views about the use of push notifications for behavioural maintenance. The framing of messages in notifications was important to participants who warned against instructional language and advocated for motivating messages that nudge people to use the app.

### Strengths and limitations

Qualitative research involving patients in dermatology is lacking (Foster et al., 2022), including in the context of developing and testing digital psychological interventions (Hewitt, Ploszajski, et al., 2022). Basing digital interventions on established theories and techniques for behaviour change helps to maximize their effectiveness (Michie et al., 2017). A key strength of this study is that it followed the PBA (Yardley et al., 2015) and utilized qualitative methods to build on our systematic review and address the gaps identified, using a larger sample than many studies identified in the systematic review and representing 12 skin conditions (Hewitt, Ploszajski, et al., 2022). Although, the use of non‐probability sampling methods arguably introduced bias (Coulson, 2015) and those who volunteered to participate may not represent the wider population.

In accordance with recommendations for dermatology research (Heague et al., 2022), this study received input from PPI contributors. Six people with lived experience of eczema, rosacea, psoriasis and X‐linked ichthyosis helped to develop the interview topic guide, and promote the study. Data collection and analysis were underpinned by established psychological models, allowing for the detailed exploration of factors that might influence intervention access and engagement (Michie et al., 2017).

There were advantages and disadvantages to using online methods. Hosting the group interviews on Zoom arguably afforded the opportunity for people from different countries to participate and increased the global reach of this study (Coulson, 2015). However, most participants were from the UK and reported having vitiligo or psoriasis, limiting the transferability of these findings. Advertising on social media platforms provided a free and efficient method of recruitment (Coulson, 2015), but several suspicious requests for participation were received and validating the identity of potential participants proved challenging, raising questions around the safety of all involved (Hewitt, Purcell, & Bundy, 2022).

### Implications for research

This study highlights several areas for future research. It seems children and young people, and adults with late condition onset, may be more in need of support to adjust to skin conditions. Qualitative research with these groups is needed to identify their specific needs and develop dedicated interventions accordingly.

The credibility of intervention developers was deemed important and scepticism towards industry‐funded interventions may be a barrier to use (Sangers et al., 2021). Qualitative research is required to better understand these concerns and find solutions to encourage engagement with interventions that are funded by, or developed in partnership with, industry.

Despite the high global prevalence of acne (Heng & Chew, 2020), support for people with acne is lacking (Ip, Muller, Geraghty, Platt, et al., 2021) and recruiting people with acne proved challenging. Accessing this group is important to ensure the app has relevance for them.

Once developed, MiDerm could serve as a useful clinical communication and decision‐making tool, but research is needed to determine whether health professionals in dermatology share this sentiment.

This study is an example of a theoretical, evidence‐based and systematic approach to developing complex digital interventions and demonstrates how qualitative research can inform development. It is, arguably, a model on which new interventions in dermatology should be based.

### Practical implications

People with skin conditions are increasingly relying on the internet and online support groups for informational and emotional support (Thorneloe, 2019). Participants concerns about the lack of evidence‐based advice and guidance, and poor regulation and censoring of freely available content that is potentially harmful, highlights an urgent need for tighter moderation and increased resourcing to protect people accessing support online.

This study shows that the idea of MiDerm is mostly acceptable to our sample. It highlights types of informational, emotional and peer support that some adults desire to live well with a skin condition and how the app content, functionality and design can facilitate self‐determination in relation to the self‐management of skin conditions. Developing the app does not negate the need for face‐to‐face psychological and peer support, and increasing the availability of, and timely access to, psychological services should remain a priority in dermatology.

### Conclusion

Common and rare skin conditions carry a substantial psychological burden, yet current service provision does not adequately meet the needs of patients, who often resort to seeking support from other, potentially less credible, sources outside of the healthcare system. This study provides a steer for content and evidence for the prospective acceptability of the MiDerm app as a medium for delivering psychological support to adults with skin conditions, but not as a replacement for face‐to‐face support. We have since developed the MiDerm app based on these findings. The next step is to test the effect of MiDerm on health outcomes.

## AUTHOR CONTRIBUTIONS

**Rachael M. Hewitt:** Project administration; conceptualization; methodology; data curation; formal analysis; writing – review and editing; writing – original draft; investigation. **Carys Dale:** Project administration; writing – original draft; writing – review and editing. **Catherine Purcell:** Conceptualization; methodology; writing – review and editing; data curation; supervision. **Rachael Pattinson:** Writing – review and editing; methodology. **Chris Bundy:** Conceptualization; methodology; writing – review and editing; data curation; supervision.

## Supporting information


Data S1.

Data S2.

## Data Availability

The data that support the findings of this study are available from the corresponding author upon reasonable request.
