# Supplementary material for: A qualitative exploration of the prospective acceptability of the MiDerm app; a complex digital intervention for adults living with skin conditions
Source: Br J Health Psychol. 2025 Jan 5;30(1):e12778. doi: 10.1111/bjhp.12778 (PMC11701206; doi:10.1111/bjhp.12778)
Supplement: Supplementary file 1 — Data S1. [file BJHP-30-0-s001.docx]

**Supplementary Material 1 – Reflexivity**

The group interviews were conducted by RH, a female PhD researcher with expertise in health psychology and the psychological impact of long-term skin conditions. RH also led on the analysis of qualitative data, with support and supervision from a team with expertise in behavioural science and psychodermatology.

There are several ways in which the researchers knowledge, views and experiences could have impacted this research. Given that this study formed part of a PhD to develop a new complex digital psychological intervention for adults with skin conditions, there may be a bias in reporting findings that support the aims of this research project. The interview topic guide covered a range of health behaviours, but the discussions that took place in the group interviews may have focused more on physical activity and dietary behaviours than other health behaviours due to RH’s personal interest in these topics. RH’s interest in women’s health may have also influenced how certain data were analysed and the conclusions that were drawn about women being potentially more vulnerable than other people with skin conditions. Conversely, personal views, experience and passion for these health behaviours could have influenced the study in a positive way as groups were able to have in-depth discussions on these topics and notice nuances and patterns in the data.

Attempts were made to offset these biases, including the supervisory team verifying conclusions and checking the strength of the language used to report the findings, but the potential biases and influences cannot be overlooked.  Field notes were completed following each interview to reflect on the interview, note any initial thoughts and reflect on reflexivity.

In addition, RH previously completed introductory and intermediate training courses in MI and has since coached other health professionals in this as both a style of communication and structure for leading consultations with patients (Hewitt et al. 2021). MI forms the basis of our clinical and academic work and RH’s expertise likely had a positive influence on how they conducted the group interviews.

**Supplementary Material 2 – Description of Theoretical Frameworks**

*The Common Sense Model of Self-Regulation (CSM) (Leventhal et al. 1997)*

The CSM is an established and evidence-based psychological model, which posits that personal illness perceptions, otherwise known as beliefs about illness, drive emotional reactions and behavioural responses to illness. It specifies five cognitive representations of illness, which, together, form one’s overall perception of illness:

- Identity – how one personally defines their illness in terms of the label they assign to it and the symptoms they associate with it.
- Cause – factors that are perceived to cause or trigger illness.
- Controllability and curability – the degree to which an individual believes their actions can influence their illness or it can be controlled using treatments, and whether an individual believes their illness can be cured.
- Consequences – the perceived impact of illness on the individual and their life.
- Timeline – beliefs about the duration of illness, for example, whether it is acute, chronic or cyclical in nature.

The CSM is dynamic in that it explains how an illness is appraised can impact on the emotional representations a person associates with their illness and their behavioural response to it. The actions one takes to address their illness are then appraised and this creates a feedback loop which can reinforce or alter existing illness beliefs and, in turn, self-regulation.

The CSM also incorporates the concept of congruence, focusing on (in)consistency between illness beliefs and approaches to management. In addition, the CSM model accounts for the social context, encouraging consideration of how social factors might influence personal beliefs, emotions and behaviour related to illness.

The CSM provides a multi-level conceptual framework for understanding, investigating and predicting the psychological processes (cognitions, emotions and behaviour), and the relationships between them, that are relevant to illness self-management.

What adults believe about their skin condition(s) is likely to affect how they feel about their condition and respond by managing it. Therefore, the CSM was be used to understand adults’ perceptions of skin conditions and how these influence their emotional reactions and behavioural approaches to self-management.

*The Theoretical Framework of Acceptability (TFA) (Sekhon et al. 2017)*

Acceptability is a concept which, broadly speaking, refers to the appropriateness of a health intervention. Acceptability should be considered across the stages of intervention development and evaluation because how appropriate an intervention is in the eyes of the people who will deliver or receive it is likely to influence the degree of engagement and its effectiveness.

Sekhon, Cartwright & and Francis (2017) theorised the concept of acceptability to support a standardised approach to defining, investigating and measuring the acceptability of health interventions and their related components. They developed the TFA which defines acceptability as:

*“A multi-faceted construct that reflects the extent to which people delivering or receiving a healthcare intervention consider it to be appropriate, based on anticipated or experiential cognitive and emotional responses to the intervention”*

The TFA states that how acceptable an intervention is depends on seven domains, including:

1. Ethicality – how aligned the intervention is to one’s personal values.
2. Affective attitude – personal feelings towards participating in an intervention.
3. Burden – the effort required to engage with an intervention.
4. Opportunity costs – benefits, profits or values that are forgone to participate in the intervention.
5. Perceived effectiveness – whether the intervention is perceived to have achieved its purpose.
6. Self-efficacy – self-confidence to perform the behaviours required for the intervention.
7. Intervention coherence – personal understanding of the intervention and how it works.

According to Sekhon, Cartwright & Francis (2017), the TFA can inform quantitative and qualitative investigations of intervention acceptability from initial development through to evaluation and implementation, and can facilitate the exploration of three types of acceptability, including the:

1. *Prospectiveerceived* acceptability of an intervention that has not yet been received or does not yet exist.
2. *Concurrent* acceptability of an intervention as determined during its delivery or use.
3. *Retrospective* acceptability of an intervention as determined following its use or completion.

The TFA has been used in the present research to assess one type of intervention acceptability; it informed the exploration of the perceived acceptability to determine whether adults with skin conditions consider the idea of delivering psychological support via a smartphone app appropriate.

*Capability Opportunity Motivation-Behaviour Model (COM-B) (Michie et al. 2011)*

The Behaviour Change Wheel (BCW) is an established and evidence-based framework for systematically designing behaviour change interventions (Michie et al. 2011). The BCW specifies nine intervention types and seven policy categories that could aid the design and implementation of new interventions. The BCW offers a person-centred approach to designing interventions because at the heart of this framework sits the COM-B Model, a behavioural hub which stipulates that engagement in a behaviour is dependent on and, in turn, can be influenced by, three interacting conditions (Michie et al. 2011):

1. Capability.
2. Opportunity.
3. Motivation.

The term *capability* refers to people’s physical and psychological ability to perform a behaviour, usually in the form of relevant skills and knowledge (Michie et al. 2011). People also need to have the *opportunity* to perform a behaviour, including the physical and social opportunity (Michie et al. 2011). Physical opportunity refers to external factors such as personal finances and being in an environment that affords the desired behaviour (Michie et al. 2011). Social opportunity relates to the social context a person finds themselves and social factors that can influence behaviour (Michie et al. 2011). Behavioural change and maintenance are also influenced by personal motivation, the cognitive and emotional processes that underpin willingness to engage in a behaviour (Michie et al. 2011). Motivation can be reflective and involve conscious evaluative and decision-making processes, or it can automatic, arising from our emotional and instinctive responses and desires (Michie et al. 2011).

The COM-B model is commonly used to identify and understand behavioural factors that need to change in order for an intervention to be effective (West and Michie 2020). Thus, it informed this research to identify any factors that could prevent and enable adults with skin conditions from engaging with MiDerm initially and overtime, as well as the factors that could influence real-world implementation of the app. Use of the COM-B model in this research helped to ensure that any potential barriers and facilitators to performing relevant health behaviours were accounted for in the development of MiDerm. Ultimately, the COM-B Model informed the behavioural analysis that was conducted to determine which behaviour(s) MiDerm would address.

Supplementary Material 3 – Codebook including

| **Theoretical concept** | **Codes and sub-codes** | **Description** |
| --- | --- | --- |
| **Theoretical Framework of Acceptability (Sekhon et al. 2017)** | | |
| Affective attitude |  | "How an individual feels about the idea of the intervention or a particular aspect of it." |
| Burden |  | "The perceived amount of effort that is required to participate in an intervention or aspect of it." |
| Ethicality |  | "The extent to which the hypothetical intervention or aspects of it fit with an individual's value system." |
| Intervention coherence |  | "The extent to which a person understands the intervention and how it works." This may be evident in personal descriptions of the app content, design, function and purpose. |
| Opportunity costs |  | "The extent to which benefits, profits or values must be given up to engage in the intervention." |
| Perceived effectiveness |  | "The extent to which the intervention or aspects of it are perceived to be likely to achieve their purpose." |
| Self-efficacy |  | "Confidence in oneself to perform the behaviour(s) required to use the intervention." |
| **Analytical framework on the impact of dermatological conditions (Pattinson et al. 2022)** | | |
| Cognitions |  | Beliefs about cause, identity, controllability and curability, consequences and timeline of skin conditions (Leventhal et al. 1984). |
|  | Cause | Beliefs about perceived or actual causes of skin conditions, including triggers. |
|  | Original cause | Factors that people believe to be the root cause or underlying mechanism that resulted in the development of their skin condition. |
|  | *Biological* | Belief that there is a biological reason for the skin condition such as a poor immune system. |
|  | *Curse* | The individual believes that they have the skin condition because they are cursed, it is their kismet or karma, or a punishment. |
|  | *Environmental* | Belief that environment factors (e.g. allergens, lace, pollution) cause the condition. |
|  | *Genetic* | Belief that the skin condition is genetic or hereditary. |
|  | *Health risk* | Belief that the skin condition occurred as a result of a predisposition (i.e., the skin condition is a known or unknown comorbidity of another condition they have) or by engaging in risky behaviours (e.g., unhealthy lifestyle behaviours). |
|  | *Lifestyle* | Belief that lifestyle factors (e.g., poor diet) cause skin conditions. |
|  | *Self* | Belief that the individual caused the skin condition because they lacked something in their worth or character including self-blame. |
|  | *Stress* | Belief that the skin condition occurred as a result of acute or prolonged psychological, social, financial, or physical stress. (Stress referring to a state where people felt that the demands of a situation exceeded their ability or resources to cope with it). |
|  | *Unknown* | The individual doesn’t know what caused the skin condition. |
|  | Triggers | Factors perceived to exacerbate skin conditions. |
|  | *Environmental* | Belief that environment factors (e.g. allergens, lace, pollution) cause symptoms to flare. |
|  | *Food* | Belief that certain foods causes symptoms to flare. |
|  | *Mood* | Belief that certain moods or changes in mood trigger the skin condition (not including stress). |
|  | *Stress* | Belief that stress causes symptoms to flare. |
|  | *Topical agents* | Belief that products (not treatments) that are applied to the skin or hair cause symptoms to flare. If the products are treatments treatment efficacy should be considered instead. |
|  | Consequences | The believed consequences of the condition, including the physical, psychological, social, financial or daily impact that they have. This captures the degree of impact too (minor, moderate or major) and could refer to broader impacts of a condition beyond the individual (i.e., to friends, family and colleagues). |
|  | General impact | The ways in which having a dermatological condition impacted aspects of daily life and functioning. |
|  | *Career* | The effect of having a skin condition on a person’s career. |
|  | *CLCI* | A person's life course has been affected by them having a skin condition. |
|  | *Daily routine* | Descriptions of how the skin condition directly or indirectly impacts one's daily routine or activities such as the ability to drive, get groceries etc. |
|  | *Dependence* | Relying on others to help with everyday tasks. |
|  | *Employment discrimination* | Comments indicating that an individual has been treated differently by others in the workplace (e.g., employer or colleagues) because of having a skin condition. |
|  | *Everyday choices* | The condition influences a person’s choices (e.g., choice of clothes, hairstyle or products). |
|  | *Expenses* | Costs the individual with the skin condition incurs as a result of their skin condition. This may include paying for prescriptions or over the counter medication, cost of new clothes or treatments etc. |
|  | *Leisure* | The skin condition directly or indirectly effects whether the person can participate in leisure activities, such as sports or going on holiday. |
|  | *Life choices* | Skin conditions influencing a person’s life choices (e.g., having a family, career). |
|  | *Loss of earnings* | The loss of earnings or wages due to living with a skin condition. |
|  | *Study* | Education or studies are affected due to having a skin condition. |
|  | *Treatment consequences* | References to treatment causing practical problems; for example, by taking up time or being messy. |
|  | Physical impact | Primary and secondary physical impacts of the skin condition, including symptoms and physical consequences that are not symptoms (respectively). |
|  | *Fatigue* | Consequences of the physical (e.g., changes bandages) and mental effort (e.g., hypervigilance, planning) required to manage condition. |
|  | *General health* | Skin condition impacts on a person’s general health. |
|  | *Mobility* | Impaired mobility, dexterity, hearing or vision due to having a skin condition. |
|  | *Pain* | Comments reflecting experiences of physical discomfort, soreness or irritation as a result of having a skin condition. |
|  | *Sensitivity* | References to the skin being sensitive to external factors, for example, to touch or when exposed to light or changes in temperature. |
|  | *Sleep disruption* | References to the skin resulting in poor sleep quality or a lack of sleep. |
|  | *Symptoms* | Physical symptoms associated with skin conditions. |
|  | Psychological impact | Cognitive and emotional consequences of having a skin condition. |
|  | *Attractiveness* | Perception that the skin condition has altered how attractive the individual is. |
|  | *Belonging* | Feeling different from other people and the importance of being part of a group or community. |
|  | *Concentration* | Expressions of finding it hard to concentrate, maintain focus or pay attention to detail. This is acknowledged as a consequence of the condition not a symptom of the condition. |
|  | *Confidence* | Lacking, or a changed sense of, self-confidence due to having a skin condition. |
|  | *Disgust* | Feels of disgust towards the self or the skin. |
|  | *Feeling exposed* | Perception that the skin condition draws unwanted attention. |
|  | *Identity* | Comments which indicate a person has absorbed their skin condition into their identity or concerns that other people define them by their skin condition. |
|  | *Loneliness* | Feeling lonely or alone. |
|  | *Loss of control* | Feeling out of or a loss of control because of a skin condition. |
|  | *Paranoia* | Comments about feeling like people are looking at them or talking about them. This code is different from other people’s reactions because it is not necessarily true but is how the person feels. |
|  | *Pressure* | Perception or examples of feeling pressure to perform, behave or appear as people without a skin condition do. |
|  | *Rejection* | Feeling or being rejected by others. |
|  | *Resent* | Feelings of resent towards skin condition. |
|  | *Resilience* | Belief that living with a skin condition has made the individual strong, able to handle burdens and recover from them. |
|  | *Self-conscious* | Comments about feeling self-conscious. |
|  | *Self-identity* | Comments about changes in identity. For example, having a skin condition changes the person or how they express themselves. |
|  | *Stress* | Reference to stress or feeling unable to cope or respond to threats or demands. Participants may use stress and distress interchangeably so don’t rely only on the word stress. |
|  | *Uncertainty* | Feeling confused or uncertain about the skin condition. |
|  | Social impact |  |
|  | *Discrimination* | Discrimination follows stigma and is the unfair or unjust treatment of an individual. |
|  | *Dismissal* | Feeling dismissed or abandoned by others or the healthcare system. |
|  | *Impact on others* | Perception that the skin condition has had an impact of the individual’s friends, family, colleagues etc. |
|  | *Initiating social relationships* | Difficulty initiating new acquaintances or friendships. In |
|  | *Intimacy* | How the condition affects a person’s ability or opportunity to be intimate with others. |
|  | *Other people's reactions* | How other people react to a person with a skin condition (e.g., stares, comments, questions, assumptions, bullying or discrimination). This also covers how people with skin conditions respond to others’ reactions. |
|  | *Relationships* | Having a skin condition has negatively impacted on close relationships, for example, with family members. |
|  | *Romantic relationships* | Having a skin condition affects a person’s ability to form new relationships, expectations of relationship or causes a breakdown in relationships. |
|  | *Social anxiety* | Worry or anxiety experienced during social situations. |
|  | *Social isolation* | Lack of contact, physically or socially, with others whether voluntarily or involuntarily. |
|  | *Social life* | Having a skin condition negatively affects a person’s social life. |
|  | *Social roles* | References to the challenges associated with performing roles important to me (e.g., to be caregiver, parent, partner, employee or student). |
|  | *Vulnerability* | Difficulty getting close to or being vulnerable around other people. |
|  | Identity | The name of a skin condition and the symptoms people associate with it. |
|  | Altered sensation | Changes in how the individual experiences touch and/or temperature; for example, being extremely sensitive and responsive to hot and cold. |
|  | Colour | Comments about the skin changing colour or redness. |
|  | Comorbidities | Comorbidities associated (or perceived to be associated) with the skin condition e.g., arthritis listed as a symptom of psoriasis, skin cancer. |
|  | Discomfort | Experiencing physical discomfort. |
|  | Disfigurement | Descriptions of altered appearance, particularly of the skin, nails or hair. |
|  | Dry skin | Comments about skin being dry. Ashy is a synonym used for dry skin particularly by people of colour. |
|  | Fatigue or loss of energy | Describing tiredness, exhaustion, lack of energy or weakness, particularly if this isn’t alleviated by sleep. |
|  | Hair loss | Comments about loss of hair or balding. |
|  | Hot or burning | Descriptions of a burning sensation in the skin or body. |
|  | Itch | The sensation and physical act of itching. |
|  | Oily skin | Comments about oily skin. |
|  | Pain | Painful sensation in affected areas or other areas. |
|  | Peeling | Descriptions of skin peeling. |
|  | Pigmentation | Descriptions of loss or changes in pigmentation including the development of white patches. |
|  | Plaques or lesions | Dry or inflamed plaques or lesions |
|  | Pompholyx | Descriptions of pompholyx: tiny itchy blisters that may weep fluid – in people with eczema. |
|  | Rashes | Descriptions of rashes may include scaly, bumpy, itchy, or otherwise irritated. Rashes are distinct from plaques or lesions which are generally demarcated. |
|  | Rough or scratchy skin | Descriptions of skin being rough or scratchy (not in terms of itch but rather in the texture of the skin). |
|  | Sensitivity to the sun | The skin condition causes sensitivity to the sun. |
|  | Severity | Description of severity of condition (e.g., mild, moderate, severe). |
|  | Spots | Comments about spots. |
|  | Weeping | Fluid leaking from affected areas (e.g., wounds, burns, blisters). |
|  | Controllability or curability | Whether something can be done to control or cure the condition. This includes person control (the influence of the individual and their actions on their condition and decisions around treatment) and treatment control (the influence of treatments on the condition and clinical outcomes). In addition, do people feel that they can control how much their condition affects their life. |
|  | Personal control | Beliefs that a person can influence their own skin condition. IPQ-R (Moss-Morris et al. 2002) items for personal control subscale:   - There is a lot which I can do to control my symptoms. - What I do can determine whether my illness gets better or worse. - The course of my illness depends on me. - Nothing I do will affect my illness. - I have the power to influence my illness. - My actions will have no effect on the outcome of my illness. |
|  | *Lack of trust in body* | The belief or feeling that their body cannot be trusted. |
|  | Self-efficacy | Confidence a personal has in their ability to influence their condition. |
|  | Treatment control and outcome expectations | The belief that treatment can influence a skin condition. IPQ-R (Moss-Morris et al. 2002) items for treatment control scale:   - There is very little that can be done to improve my illness. - My treatment will be effective in curing my illness. - The negative effects of my illness can be prevented (avoided) by my treatment. - My treatment can control my illness. - There is nothing which can help my condition. |
|  | *Decisions about healthcare* | The extent to which the individual believes they were able to make decisions about their care including treatments. |
|  | *Treatment consequences* | Side effects (positive or negative) of the treatments available or actually taken. |
|  | *Treatment efficacy and choice* | Perceptions of treatment efficacy and the range of treatments available. |
|  | Timeline | Beliefs relating to the trajectory of a skin condition (e.g., acute, chronic, cyclical). Includes beliefs and expectations about the duration of a condition, and the variability and predictability of symptoms. |
|  | Duration | Time since onset of condition. |
|  | Flares | The sudden appearance or worsening of symptoms. |
|  | Life stages | How the impact varies, in severity or type, across life stages. |
|  | Progression | How the condition is expected to progress, including improvements, deterioration and the development of comorbidities. |
|  | Symptom variability or predictability | The variability or predictability of the condition. |
|  | Trigger variability or predictability | The variability or predictability of the triggers of the condition. |
| Emotions |  | Affective responses to the skin condition and evaluation of the (potential) emotional impact of the condition. Positive, neutral and negative emotional responses. |
|  | Negative | • Participant thoughts or comments about illness, awareness of having the illness, or reminders about illness that trigger negative emotions. • Feelings of depression or low mood, being upset, irritated, angry, afraid, embarrassed, ashamed or anxious in any way. |
|  | Anger |  |
|  | *Betrayal* | Feeling that their body has betrayed them. |
|  | *Frustration* | Feeling that their will or goals are being denied. Can be related to the skin condition (e.g. frustration about having the skin condition, frustration with flares or treatments) and to other phenomena such as frustration with clinicians or others knowledge of the skin condition. |
|  | *Hostility* | Feeling or expressing unfriendly or aggressiveness towards themselves or others, for example, being rude to clinicians. |
|  | Annoyed or irritated | Feelings of annoyance or irritation. |
|  | Anxiety and fear |  |
|  | *Anticipatory worry* | Anxiety regarding possible or future experiences or situations related to having a skin condition. |
|  | *Clinician exaggeration* | Fear or anxiety results from information, sometimes exaggerated, provided by clinicians. |
|  | *Fear* |  |
|  | Current and future morbidities | Expressions of feeling fearful or anxious regarding current comorbidities or development of them in the future. |
|  | Economic | Fear of financial loss or not being able to manage financially as a result of the skin condition. |
|  | Genetic | Fear of the condition being hereditary and ‘passing the gene’ on to children. |
|  | Medical consequences | Fear of negative consequences of medication or treatments. |
|  | *Guilt* |  |
|  | Brought on | Feelings of guilt regarding the skin condition. This could be feeling guilty for bringing the skin condition into the family and perceived or real consequences of that (e.g. family ostracised by community). |
|  | Parent | Feeling guilty that they have passed the skin condition on to their children. |
|  | Survivor guilt | Feeling guilty that their skin condition is less severe or has fewer consequences (e.g. death) than that of others with the same skin condition or other conditions. |
|  | *Hypervigilance* | Descriptions of a heightened sense of alertness with regard to the skin condition. Frequent thinking, planning and engaging in behaviours to manage the skin condition. |
|  | *Perceived worry* | Worrying that other people will worry about them because of the skin condition. |
|  | *Preoccupation, rumination or obsession* | The person expresses that they spend a great deal of time thinking about the skin condition or ruminating which has a negative impact. |
|  | Desperation | Comments or alluding to being desperate for something. Often a cure or effective management. |
|  | Distress | Comments about being distress, defined here as depression and anxiety or general negative emotions, rather than specifically depressed or anxious. Participants may use stress and distress interchangeably so don’t rely only on the word distress. |
|  | Emotional pain or hurt | References to feeling emotional pain or hurt. |
|  | Jealousy |  |
|  | Low mood and depression |  |
|  | *Depression* | Reference to depression. |
|  | *Hopeless or helpless* | Negative feelings towards the future, and feeling that nothing can be done to influence the condition. |
|  | *Lack of self-efficacy* | The individual expresses that they lack or do not have confidence in their ability execute behaviours to meet certain goals unrelated to the skin condition (i.e. not personal control) as a result of the skin condition. |
|  | *Lack of self-esteem* | An unfavourable attitude towards one’s self. The individual may express feelings of low self-worth or value or negative appraisals of their own appearance, beliefs, emotions and behaviours. |
|  | *Low mood* | Expressions of low mood, feeling low in spirits or a general loss of interest in things. |
|  | *Low motivation* | Reference or examples of lack of motivation. |
|  | *Sad* | Feelings of sadness |
|  | *Self-harm* | Intentionally damaging or injuring one’s self. |
|  | *Suicidality* | Suicidal ideation (serious thoughts about taking one's own life), suicide plans and suicide attempts. |
|  | Shame and embarrassment |  |
|  | Shock or surprise | Comments about feeling shocked or surprised about the skin condition. |
|  | Neutral | Participant thoughts or comments about illness, awareness of having the illness, or reminders about illness do not trigger psychological coping mechanisms (e.g., worry) or any emotions. |
|  | Positive | Thoughts or comments about illness, awareness of having the illness, or reminders about illness that trigger positive emotions. |
|  | Empowered | A skin condition, or how a person copes with their skin condition, makes them feel empowered. |
|  | Gratitude or appreciation | Feeling grateful or appreciative because of the skin condition. |
| Coping |  | The cognitive, emotional and behavioural process of managing and adjusting to the skin condition in an attempt to return to normal functioning (Lazarus and Launier 1978). Coping strategies described and measured by the most commonly used coping measures, COPE (Carver 1997) and the Ways of Coping Checklist (Folkman and Lazarus 1988) are:   - Active coping (e.g., ‘I’ve been taking action to try to make this situation better’). - Planning (e.g., I’ve been trying to come up with a strategy’). |
|  | Approach coping strategies | Confronting the problem, gathering information, and taking direct action (Roth and Cohen 1986).  Approach strategies allow for appropriate action, and noticing and taking advantage of opportunities to make the skin condition more controllable (Roth and Cohen 1986). |
|  | Active coping |  |
|  | *Advocacy* | Participating in advocacy work either formally (e.g., through patient organisation) or informally (e.g., answering questions, educating others). |
|  | *Cover, conceal or camouflage* | Developing strategies to conceal the skin condition so it’s not visible to allow the individual to return to normal functioning. |
|  | *Enhancing wellbeing* | Activities to enhance wellbeing, feel calm, stress reduction. |
|  | *Healthy behaviours* | Engaging in healthy behaviours, such as eating healthily, taking physical activity, reducing smoking and not drinking alcohol or taking drugs. |
|  | *Seeking information for self-management* | Actively seeking information for how to manage the skin condition. |
|  | *Seeking information for understanding* | Researching to better understand the condition. |
|  | Hope | Reference to hope or optimism as a coping strategy. |
|  | Humour | Making light of or making jokes about the skin condition. |
|  | IDGAF | Comments about not caring what people think of them anymore and this allows them to cope better. |
|  | Planning | References to making plans |
|  | Positive reframing | Reframing the skin condition and living with the skin condition in positive terms. |
|  | Religion | Turning to faith as a way of coping |
|  | Stages of coping | Describing a journey or timeline of stages of coping. Sometimes compared to stages of grief. |
|  | Using emotional support | Seeking out others for emotional support, not necessarily people with lived experience of skin conditions. |
|  | *Looking for people with shared experience* | Seeking out other people with skin conditions for emotional and instrumental support. |
|  | Avoidance coping strategies |  |
|  | Behavioural disengagement | Withdrawing from normal behaviours |
|  | *Hiding* | Developing strategies to hide the skin condition that negatively impact the individual, such as self-isolation, wearing unattractive clothes. |
|  | *Limiting challenges* | Not engaging in anything deemed a challenge, remaining in comfort zone at all times, in an attempt to reduce emotional impact. |
|  | *Limiting choices* | Allowing the skin condition to determine or limit choices such as where to socialise in an attempt to reduce emotional impact. |
|  | Denial | Reference to denying one's condition |
|  | Distancing |  |
|  | *Lack of engagement with healthcare* | Being uninterested in or unwilling to appraise their health or to engage in healthy behaviours. |
|  | Escape or avoidance | Trying to escape or avoid a skin condition. This might be avoiding seeing the skin (e.g., covering mirrors) or thinking about the condition or escaping the feelings and reality of having a skin condition. |
|  | Sarcasm | Use of sarcasm when talking about the skin condition. |
|  | Self-control | Trying to control thoughts, feelings and behaviours related to condition. |
|  | Self-distraction | Actively choosing to think about other things or performing behaviours that allow a person to take their mind away from their condition. |
|  | Substance use | Reference to using alcohol or drugs. |
|  | Unhealthy lifestyle behaviours | Engaging in unhealthy behaviours, such as poor diet, smoking or a lack of exercise, with the view to mitigate the impact of the skin condition (e.g., not exercising to avoid itching). |
| Other |  |  |
|  | Acceptance | Comments about accepting a skin condition. |
|  | Time | Coming to accept a condition with the passing of time. |
|  | Awareness | General awareness of skin conditions among patients, families, health care services and society. A potential mediator/moderator of impact. |
|  | Medical awareness | The need for better awareness or knowledge of skin conditions in the medical community (e.g., lack of research). |
|  | Before and after | Comments about life before and after the onset of the condition. |
|  | CLCI | Evidence of CLCI or MLCDs. |
|  | Dirty or clean | This code is used to track comments about dirty and clean so that we can see reflect on what this might mean and where it fits later. |
|  | Individual differences | Individual differences or personality traits as a mediator/moderator of impact. |
|  | Normal | Comments about what is “normal”. This can be from the participants’ point of view of normal or how other people perceive normal. |
|  | Services |  |
|  | Clash of beliefs | The meeting of opposing beliefs regarding the skin condition. Example: clinicians express a medical belief that is at odds with the cultural beliefs about the skin condition. |
|  | Diagnosis | References to diagnoses of a condition. |
|  | *Accuracy* | Discussing how accurately clinicians have been able to diagnose the skin condition. For some, they might have had multiple, incorrect diagnoses. |
|  | *Timing* | Discussing the timing of the diagnosis of the skin condition. Particularly in reference to how quickly a diagnosis is made following symptom onset and the consequences of the time difference between onset and diagnosis. For example, in terms of severity, progression and variability of symptoms. |
|  | Equity of access | Discussing to what extent equity of access to affordable, high quality, culturally and linguistically appropriate care in a timely manner exists and whether/how social, economic, demographical, or geographical factors interact with this. |
|  | Individual | Factors related to services on the indiviudal level. |
|  | *Access to treatment* | Whether the person is personally able to access treatment. For example, whether they are physically capable of attending medical appointments due to disability. (This may be perceived to have consequences for the progression of the condition) |
|  | Information provided by health professionals | Comments about the quality of the information given by clinicians. For example, references made clinicians focusing information only on medical treatments and not discussing triggers. |
|  | Lack of engagement with decision making | Discussing the extent to which clinicians involve or share decision making with people with skin conditions. For example, whether the patient is able to be involved in choosing which treatment is administered or treatment goals. |
|  | Scepticism | A lack of trust or scepticism of services or clinicians. For example, a belief that clinicians purposely behave and manage the condition in a way that brings continued income. |
|  | System |  |
|  | *Availability of additional support* | References to availability of psychological, emotional, mental health support or other types of support related to the management of skin conditions. |
|  | *Availability of standard medical care* | The extent to which services (e.g., access to dermatologist, treatment etc) are available to people with skin conditions. |
|  | *Organisation of care* | How, and the extent to which, care is co-ordinated with other services; for example, how well dermatology and rheumatology services are co-ordinated for people with psoriatic arthritis. |
|  | Treatment |  |
|  | *Discrepancies in goals, outcomes and expectations* | Expressions of differences between clinician and patient expectations, goals and appraisal of outcomes. |
|  | Social support | The extent to which friends and family support a person with a skin condition. Social support may be a mediator or moderator of impact. |
|  | *Being known* | A code to track comments about feeling that the experience of having a skin condition is easier around people they already know as opposed to strangers. |
|  | Tough, struggle or battle | This code is used to track the use of this language so that we can see reflect on what this might mean and where it fits later. |
| COM-B Model (Michie et al. 2011) | | |
| Capability |  | “The individual’s psychological and physical capacity to engage in the activity concerned. It includes having the necessary knowledge and skills.” |
|  | Physical | Behaviour is influenced by the person’s knowledge of and skills to perform it. |
|  | Psychological | Behaviour is influenced by the person’s ability to engage in the necessary thought processes, including understanding the behaviour and reasoning for performing it. |
| Opportunity |  | “The factors that lie outside the individual that make the behaviour possible or prompt it.” |
|  | Physical | Behaviour is influenced by a person’s physical environment. |
|  | Social | Behaviour is influenced by a person’s social environment and the social factors that influence how they think about a behaviour (e.g., culture, social norms and language). |
| Motivation |  | “Brain processes that energize and direct behaviour, not just goals and conscious decision-making. It includes habitual processes, emotional responding, as well as analytical decision-making.” |
|  | Automatic | Behaviour is influenced by motivation that is automatic, does not require thinking about and arises from our emotions and impulses. |
|  | Reflexive | Behaviour is influenced by motivation that arises from logical thought, evaluation and planning. |
| IPQ-R, Illness Perceptions Questionnaire – Revised (Moss-Morris et al. 2002) | | |
| CLCI, Cumulative Life Course Impairment | | |
| MLCDs, Major Life Changing Decisions | | |
| COM-B, Capability, Opportunity, Motivation – Behaviour Model (Michie et al. 2011) | | |

Supplementary Material 4 – Sample Characteristics

| Categorical demographic variable | *N* | % |  |  |
| --- | --- | --- | --- | --- |
| Gender |  |  |  |  |
| Male | 13 | 30.2 |  |  |
| Female | 30 | 69.8 |  |  |
| Ethnicity |  |  |  |  |
| White | 36 | 83.7 |  |  |
| South Asian | 2 | 4.7 |  |  |
| Other | 5 | 11.6 |  |  |
| *New Zealand European/Pacific Islander* | 1 | 2.3 |  |  |
| *White and Southeast Asian* | 1 | 2.3 |  |  |
| *Anglo Caribbean* | 1 | 2.3 |  |  |
| *Mixed heritage* | 1 | 2.3 |  |  |
| *Indian* | 1 | 2.3 |  |  |
| Skin condition |  |  |  |  |
| Psoriasis | 11 | 25.6 |  |  |
| Eczema | 4 | 9.3 |  |  |
| Vitiligo | 12 | 27.9 |  |  |
| Hidradenitis suppurativa | 1 | 2.3 |  |  |
| Pachyonychia congenita | 1 | 2.3 |  |  |
| Hyperpigmentation | 1 | 2.3 |  |  |
| Ichthyosis | 9 | 20.9 |  |  |
| *Netherton syndrome* | 2 | 4.7 |  |  |
| *Ichthyosis vulgaris* | 2 | 4.7 |  |  |
| *Llamella ichthyosis* | 2 | 4.7 |  |  |
| *Epidermolytic hyperkeratosis* | 1 | 2.3 |  |  |
| *Type not specified* | 2 | 4.7 |  |  |
| Multiple skin conditions | 4 | 9.3 |  |  |
| *Post-hypertrophic cystic acne scarring, hyperpigmentation* | 1 | 2.3 |  |  |
| *Seborrheic dermatitis, psoriasis* | 1 | 2.3 |  |  |
| *Seborrheic dermatitis, vitiligo, eczema* | 1 | 2.3 |  |  |
| *Acne, rosacea, hidradenitis* | 1 | 2.3 |  |  |
| Country |  |  |  |  |
| England | 29 | 67.4 |  |  |
| Wales | 9 | 20.9 |  |  |
| Scotland | 1 | 2.3 |  |  |
| Ireland | 1 | 2.3 |  |  |
| USA | 1 | 2.3 |  |  |
| Demark | 1 | 2.3 |  |  |
| Sweden | 1 | 2.3 |  |  |
| Patient organisation member |  |  |  |  |
| Yes | 14 | 32.6 |  |  |
| No | 29 | 67.4 |  |  |
| Continuous demographic variables | Mean | *SD* | Minimum | Maximum |
| Age (years) | 44.63 | 13.02 | 25 | 69 |
| Years lived with condition* | 28.02 | 15.92 | 4 | 68 |
